# Retinal Ganglion Cells—Diversity of Cell Types and Clinical Relevance

**DOI:** 10.3389/fneur.2021.661938

**Published:** 2021-05-21

**Authors:** Ungsoo Samuel Kim, Omar A. Mahroo, John D. Mollon, Patrick Yu-Wai-Man

**Affiliations:** ^1^Kim's Eye Hospital, Seoul, South Korea; ^2^John van Geest Centre for Brain Repair and MRC Mitochondrial Biology Unit, Department of Clinical Neurosciences, University of Cambridge, Cambridge, United Kingdom; ^3^Cambridge Eye Unit, Addenbrooke's Hospital, Cambridge University Hospitals, Cambridge, United Kingdom; ^4^Moorfields Eye Hospital NHS Foundation Trust, London, United Kingdom; ^5^Institute of Ophthalmology, University College London, London, United Kingdom; ^6^Section of Ophthalmology, King's College London, St. Thomas' Hospital Campus, London, United Kingdom; ^7^Department of Psychology, University of Cambridge, Cambridge, United Kingdom

**Keywords:** retinal ganglion cell, optic neuropathies, hereditary optic neuropathies, acquired optic neuropathies, electrophysiological tests, neuro-ophthalmology

## Abstract

Retinal ganglion cells (RGCs) are the bridging neurons that connect the retinal input to the visual processing centres within the central nervous system. There is a remarkable diversity of RGCs and the various subtypes have unique morphological features, distinct functions, and characteristic pathways linking the inner retina to the relevant brain areas. A number of psychophysical and electrophysiological tests have been refined to investigate this large and varied population of RGCs. Technological advances, such as high-resolution optical coherence tomography imaging, have provided additional tools to define the pattern of RGC involvement and the chronological sequence of events in both inherited and acquired optic neuropathies. The mechanistic insights gained from these studies, in particular the selective vulnerability and relative resilience of particular RGC subtypes, are of fundamental importance as they are directly relevant to the development of targeted therapies for these invariably progressive blinding diseases. This review provides a comprehensive description of the various types of RGCs, the developments in proposed methods of classification, and the current gaps in our knowledge of how these RGCs are differentially affected depending on the underlying aetiology. The synthesis of the current body of knowledge on the diversity of RGCs and the pathways that are potentially amenable to therapeutic modulation will hopefully lead to much needed effective treatments for patients with optic neuropathies.

## Introduction

It was a clinical ophthalmologist, and an unusually interesting one, who first proposed that different fibres in the optic nerve carry different attributes of the retinal image, such as colour and spatial detail. Born in Charleston in 1830, John Julian Chisolm graduated from the Medical College of South Carolina in 1850 and gained further training on two visits to Europe (1). After the bombardment of Fort Sumter, he was commissioned into the Confederate Army and within 4 months had published the first of three editions of his “Manual of Military Surgery.” In the years after the Civil War, he specialised in ophthalmology and in 1869, he reported, in the Moorfields house journal, how form vision had recovered before colour vision in a case of neuritis, leading him to ask “…whether there are special nerve fibres, for the recognition of special colours, independent of those used in the clear definition of objects.” (2). As early as the eighteenth century, there had been suggestions that there are different retinal fibres for different colours [e.g., (3, 4)], but Chisolm's is likely to be one of the first suggestions that different *attributes* of the image—such as form and colour—are carried by different fibres.

Today, it is clear that the retina does not deliver to the brain a pixel-by-pixel representation of the pattern of light falling on the photoreceptors. There are about 120 million rods and 6 million cones, whilst the output of the retina is transmitted by around 1.2 million retinal ganglion cells. Thus, there exists significant “pre-processing” of the visual signal by the retinal neuronal layers. The retinal ganglion cells (RGCs) extract in parallel different attributes of the image—spatial contrast, colour, motion, flicker, fine and coarse textures, absolute light level—and deliver this information to different sites within the visual system (5, 6). At least 18 different types of ganglion cells are now thought to be present in the primate and human retina, all of them functionally and morphologically distinct (7, 8). The individual types gain their functional specificities in turn from dedicated circuits that lie between the photoreceptors and the ganglion cells (9). The primate retina is currently thought to include 2 types of horizontal cells, 12 types of bipolar cells and more than 25 types of amacrine cells (Table 1).

**Table 1:** The number of types of photoreceptors, bipolar cells, and retinal ganglion cells in different species.

	**Mouse**	**Cat**	**Rabbit**	**Primates**
Photoreceptors	3 (one rod and S- and M- cones) (10)	3 (one rod and S- and M- cones)	3 (one rod and S- and M-cones)	4 (one rod and three cones)
Bipolar cells	~15 (11)	~9 (12)	~13 (13)	~12 (14)
Retinal ganglion cells	~30 (5)	~23 (12)	~20? (15)	Up to 18

Our purpose in this review is to provide clinicians with a brief survey of the different types of ganglion cells to highlight the possibility of either selective impairment or selective survival of particular types of cells. Subsequently, we discuss both clinical and research methods for evaluating the structure and function of RGCs, and survey a number of relevant clinical conditions before briefly discussing future avenues of research. This review will focus on primate and human studies. Lower mammals appear to enjoy a richer range of ganglion cell types with 40 or more different types having been reported in the mouse (16, 17). It is certainly attractive to consider the extensive literature devoted to the mouse since a remarkable array of histological, physiological and genetic methods have become available to study murine ganglion cells over the past two decades—methods that cannot all be applied to primates. In many areas, there is wide conservation across mammalian phylogeny, not only of cell morphologies, but also of the physiological circuits that underlie the functional specialisation of particular types of cell. On the other hand, the retina of each species is well adapted to the visual theatre into which that species is born, having evolved to match the requirements of that animal's visual world and to serve the animal's survival and reproduction (18). Analogies between different species may, therefore, sometimes be misleading (19). Even between macaques and humans, there may be occasional differences in the genes expressed in otherwise corresponding types of ganglion cells (20).

“Midget” and “parasol” types comprise more than 80% of all ganglion cells in human and primate retinas (20). Given this predominance, it is tempting to neglect the many minority types, but functional importance should not be equated with relative numbers. Midget and parasol cells have relatively small dendritic fields and therefore, large numbers are needed to tessellate—to tile—the retina. Typically, the rarer ganglion cells have larger, often much larger, dendritic fields (7), and thus many fewer are needed to tesselate the entire retina. Yet, these wide-field cells may have a critical functional role, perhaps in everyday life or perhaps in unusual, but life-threatening conditions. In the case of several wide-field ganglion cells, this function is still unknown, and it may fall to an alert clinician, in the tradition of Chisolm, to detect the selective impairment that provides important clues.

### Classification of Ganglion Cells—Methods

The taxonomy and the nomenclature of ganglion cells, like taxonomies and nomenclatures in other branches of biology, have generated unexpectedly contentious debates, especially when different methods of classification give different solutions or when nomenclatures are translated from one species to another (21). We briefly survey the several techniques that have led to the current taxonomy of ganglion cells. The alternative methods can themselves be grouped into anatomical, molecular, and functional classification.

#### Anatomical Classification

The cell bodies of most ganglion cells, and the layer formed by their axons, lie close to the inner limiting membrane adjacent to the vitreous, although occasional “displaced” ganglion cells are seen among amacrine cells in the inner nuclear layer [(22, 23), p. 309]. In the central regions of the retina, there are up to eight layers of ganglion cells, whereas in the far periphery, near the *ora serrata*, there are only sparse clumps of two or three ganglion cells with gaps between them (23).

#### Morphology

**Figure 1:**
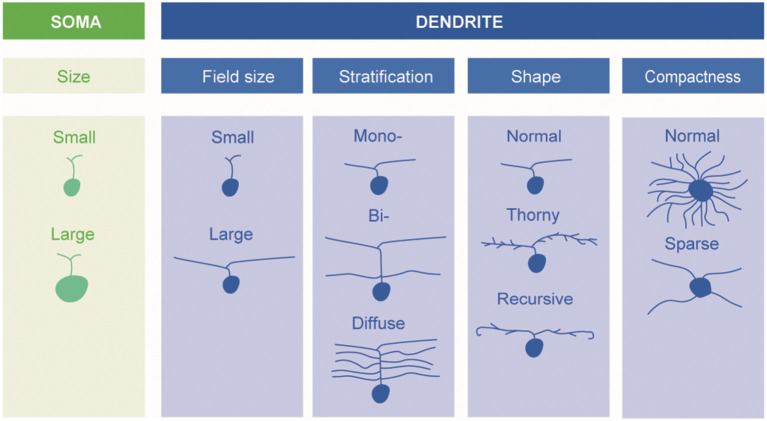
Schematic description of retinal ganglion cells. Morphological types of RGCs are classified based on soma size and dendrite morphology (*by Ungsoo S. Kim*).

A fundamental basis for classification—and the one mainly adopted in the present review—is the size and morphology of the cell body and dendrites, as well as the extent of the dendritic field (Figure 1). Already in 1893, using the silver staining method of Golgi, Cajal distinguished several types of ganglion cells by such features, but it was not until 1935 that the predominant ganglion cells of the central region of the primate retina, the midget cells, were described by Stephen Polyak (who had not yet anglicised his name) (24).

#### Upstream Connexions: The Input Circuits and the Stratification of Dendrites

The functional properties of a given ganglion cell must necessarily depend on the excitatory and inhibitory inputs it receives from bipolar cells and from amacrine cells. The many different types of amacrine cells allow the construction of specialised circuits that determine the responses of their associated ganglion cells. In principle, it would be possible for two ganglion cells of identical morphology to receive different inputs and thus differ in their functional properties.

A fundamental basis for classifying ganglion cells is therefore the stratum, or strata, of the inner plexiform layer (IPL) in which their dendrites extend. Some are “monostratified,” their dendrites confined to one stratum; some are “bistratified,” having two distinct layers of dendrites (Figure 1). The level or levels at which the dendrites of a given ganglion cell stratify are very characteristic (6). One gross division is between the inner and outer layers of the inner plexiform layers. Bipolar cells of the OFF type predominantly make contact with ganglion cells in the outer part whereas bipolar cells of the ON-type predominantly synapse in the inner part.

#### Downstream Connexions: The Projections of Retinal Ganglion Cells

**Figure 2:**
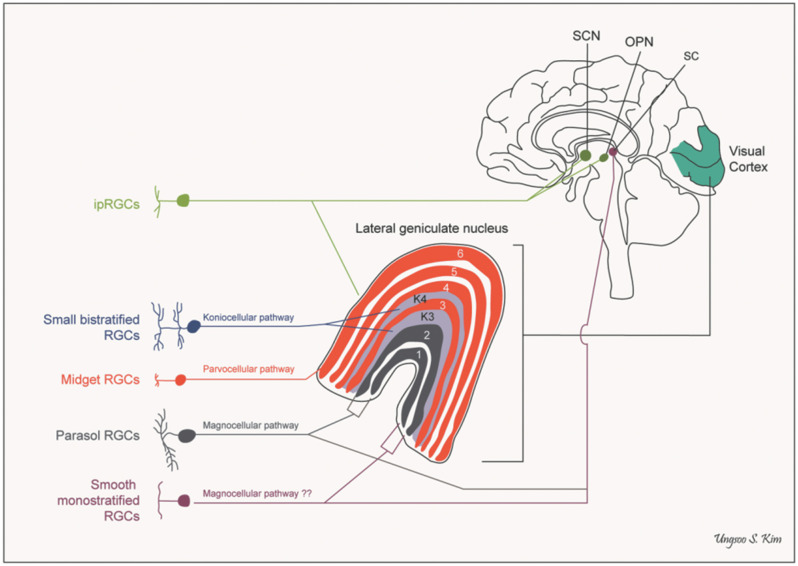
Pathways of retinal ganglion cells. OPN, olivary pretectal nucleus; RGCs, retinal ganglion cells; SC, superior colliculus; SCN, suprachiasmatic nucleus (*by Ungsoo S. Kim*).

A further anatomical classification, and one of special interest to the neuro-ophthalmologist, can be based on the projection sites of each class of ganglion cell. Besides the lateral geniculate nucleus, there are several other brain areas that receive direct projections from the retina, including the superior colliculus, the pulvinar complex, the olivary pretectal nucleus, the supraoptic nucleus of the optic tract, the paraventricular nucleus, the suprachiasmatic nucleus, and the dorsal raphe nucleus (see Figure 2) (25–27).

Classically, the projections of the ganglion cell layer could be established by anterograde tracing, e.g., by intraocular injection of a radioactive agent or cholera toxin subunit B (28), but anterograde tracing of this kind does not identify the type, or types, of ganglion cell in which the projection originates. Retrograde methods, e.g., injecting horseradish peroxidase into the central site, allow specific ganglion cells to be labelled in the retina. An example would be the study by Cowey et al. showing retrograde labelling of several types of primate ganglion cells—including parasol and midget—after injections to the pulvinar (29). One modification of the retrograde method was introduced by Rodieck and Watanabe, who used a fluorescent marker for retrograde tracing and then, in an *in vitro* preparation, injected rhodamine-conjugated horseradish peroxidase into individual cells that had been labelled by the retrograde marker—a procedure that gave better filling of dendrites than did simple retrograde tracing with horseradish peroxidase (30).

#### Molecular Classification

The Golgi method is “noted for its fickleness” (31): this is its strength, in that it exquisitely reveals isolated neurons against a pale background, but it is also its weakness, in that the random staining is unpredictable and not specific to a particular type of cell (32). What are obviously desirable, especially for quantitative purposes, are methods that label either a single class of ganglion cells or only a small number of classes. In the case of lower mammals, many molecular markers (e.g., using antibodies or gene expression) have been developed to identify individual classes (Table 2) [e.g., (46)].

**Table 2:** Antibodies used in immunohistochemistry for retinal ganglion cells.

**Antigen**	**Host species**	**Specificity**	**References**
βIII-tubulin	Mouse	Monoclonal	(33)
Islet-1	Mouse	Monoclonal	(34)
Syntaxin-1	Mouse	Monoclonal	(35)
GFAP	Mouse	Monoclonal	(36)
ED1	Rabbit	Monoclonal	(37)
Brn3a	Goat	Polyclonal	(38)
Thy1 (CD90)	Mouse	Monoclonal/Polyclonal	(39)
CaBP (DB3a)	Mouse	Monoclonal	(40)
CD15 (FMB, DB6)	Mouse	Monoclonal	(41, 42)
RBPMS	Rabbit, Mammalian	Polyclonal	(43–45)

There has been less work of this kind in primates, but of particularly note is the work by Peng et al. (19) and Yan et al. (20) who identified RNA expressed in individual cells from macaque and human retinas (Table 3). In macaque, they were able to group peripheral ganglion cells into 18 clusters and foveal ganglion cells into 16 clusters. Three of the peripheral clusters in macaque retina (and two in the case of human) expressed *OPN4*, the gene for melanopsin and thus a marker of intrinsically photosensitive RGCs (ipRGCs). Fourteen of the foveal clusters corresponded to peripheral clusters. Although a similar set of transcription factors is used in mouse and primate ganglion cells, there was little correspondence in the detailed RNA expression patterns of individual cell types. In particular, there was no clear mouse equivalent of the midget ganglion cell of the primate. The patterns of RNA expression were very similar for human and macaque retinas, but occasional differences were seen. For example, the gene *RBPMS2* was expressed in human, but not macaque midget ganglion cells. In the present context, it is significant that genes known to be associated with glaucoma were found to be predominantly expressed in ganglion cells, sometimes selectively—e.g., *SIX6* in midgets (19).

**Table 3:** Expression molecular markers of major retinal ganglion cells in primates.

**Type of retinal ganglion cells**	**Expression molecular markers**	**References**
ON-midget RGCs	*TPBG, GUCY1A3*	(47)
OFF-midget RGCs	*TBR1, GUCY1A3*	(19)
ON-parasol RGCs	*CHRNA2, SPP1, RBPMS2*	(19, 48)
OFF-parasol RGCs	*CA8, SPP1, RBPMS2*	(19, 48)
Large sparse RGCs	*SATB2*	(49)
ipRGCs^*^	*OPN4*	(50, 51)

* *ipRGCs, intrinsically photosensitive retinal ganglion cells*.

#### Functional Classification

In functional experiments, a physiological measure of a cell's response is recorded when the retina is stimulated with a specific stimulus. Psychophysical work has often guided the choice of stimulus. In the second half of the twentieth century, psychophysicists endeavoured to isolate “mechanisms” or “channels” within the visual system. These constructs were hypothetical, but the hope—not without foundation—was that they corresponded to independent neural channels. The isolation of a given channel was achieved by construction of a stimulus to which the channel might be maximally sensitive and by the use of selective adaptation stimuli to reduce the sensitivity of other channels [e.g., (52–54)]. The techniques that were honed by psychophysicists were often adopted by electrophysiologists and applied to individual ganglion cells. The same techniques often also inspired new clinical testing methods, such as frequency-doubling perimetry, designed to isolate channels with non-linear responses (55). Psychophysics has also inspired the instruments used to deliver the carefully crafted stimuli needed in electrophysiological work—Maxwellian-view optics in the 1960's, computer-controlled CRT displays in the 1980's, and digital light processors in this century.

**Figure 3:**
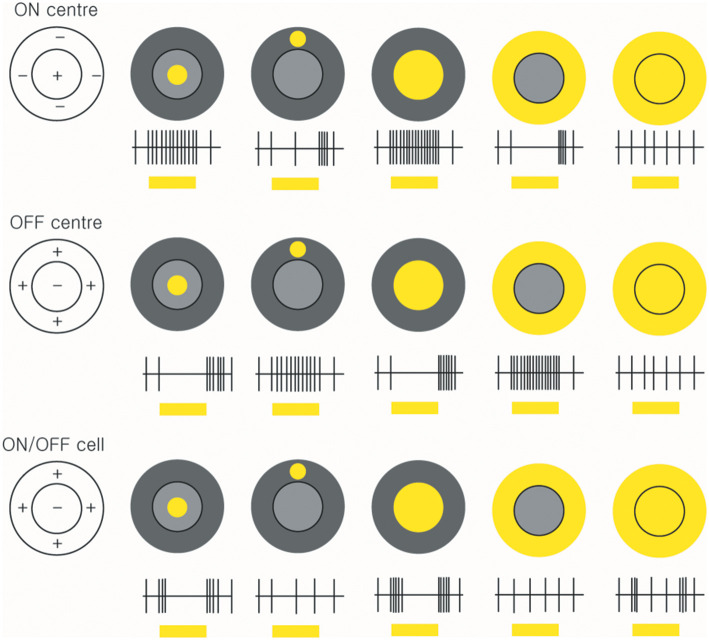
Receptive fields and responses of ON-centre, OFF-centre, and ON/OFF retinal ganglion cells. ON-centre RGCs (upper panels) increase their rate of discharge when the light illuminates in the centre. However, when the surround field is illuminated, the ON-centre RGCs are suppressed. OFF-center RGCs respond when the light turns off (middle panels). ON/OFF cells are triggered briefly when the light turns on or off (lower panels) (*by Ungsoo S. Kim*).

The celebrated study of Kuffler initiated the extracellular recording of action potentials from individual ganglion cells in the mammalian retina by means of fine-tipped microelectrodes (56). Kuffler demonstrated the antagonistic centre-surround arrangement that characterises the receptive fields of many ganglion cells: stimulation of the centre of the receptive field evokes an ON response in some cells and an OFF response in others, whereas stimulation of a surrounding region evokes the opposite response (Figure 3).

A pioneer in the study of primate RGCs was Peter Gouras, who made microelectrode recordings *in vivo* from *Macaca mulatta* and made a basic distinction between cells with transient (“phasic”) responses and those with sustained (“tonic”) responses (57). Influenced by the psychophysical work of Stiles (58), Gouras presented small monochromatic flashes of varying wavelength on monochromatic adapting fields and showed that sustained cells typically drew inputs from one class of cone in the centre of their receptive field and inhibitory inputs from other types of cone in the surround. Given the specificity of the centre input and the predominance of sustained cells in the central field, he identified them with the midget cells of Polyak. The phasic cells were more common in the periphery and appeared to draw inputs of the same sign from long- and middle-wave cones, with little input from short-wave cones.

In the last two decades, it has become possible to record concurrently from several hundred ganglion cells in an eye-cup preparation. A segment of peripheral retina, with pigment epithelium intact, can be placed with ganglion cell layer downwards on a planar array of, say, 512 extracellular micro-electrodes. In the work of Field et al. for example, the macaque retina was stimulated with a lattice of square pixels that varied randomly and independently in chromaticity (59). The responses of individual ganglion cells, identified later off-line, were correlated with the random pattern of stimulation to determine their preferred stimuli.

The introduction of adaptive-optics scanning-light ophthalmoscopy (AOSLO) combined with calcium imaging has been used to monitor the responses of individual ganglion cells in the eye of a living primate [e.g., (60)]. Action potentials cause rapid changes in intracellular free calcium and these can be revealed with a fluorescent protein calcium sensor. McGregor et al. used the sensor GCaMP6s for that purpose. While the retina was stimulated with orange (590 nm) drifting gratings, a 488-nm laser was focussed on the ganglion cell layer to excite the calcium sensor, and the fluorescence was detected in a band at 517–520 nm. A limitation of currently available calcium sensors, such as GCaMP6s, is that they have relatively large time constants (0.6 s) and so cannot follow high frequencies of modulation.

#### Combinations of Methods

A critical task has been to relate one method of classification to a second. An early success was achieved by intracellular recording with a micropipette electrode filled with a dye such as Procion yellow (61): after a basic characterisation of the cell's response, the passage of hyperpolarizing current could be used to inject the stain into the cell, for later histological examination. Nelson et al. used such a method in eye-cup preparations from cat to show the fundamental mammalian separation of the inner plexiform layer into ON and OFF sublaminae (62). Dacey and Lee used a refinement of this technique in which primate ganglion cells of specific morphology were targeted in a flat-mount preparation under visual inspection, recordings were made with an intracellular micropipette, and the cell was stained by intracellular injection of the fluorescent dye pyranine during recording (63). It was this work that first identified the small bistratified ganglion cell as carrying the excitatory signal of short-wave primate cones.

In 2003, Dacey et al. introduced a powerful technique that has been central to our modern understanding of the range of ganglion cell types (64). The method allows the morphology of the cell to be related not only to stratification level in the inner plexiform layer and to downstream projection sites, but also to the functional characteristics of the cell. Rhodamine dextran was injected *in vivo* into a central site (e.g., the lateral geniculate nucleus or the superior colliculus) and the dye travelled retrogradely to the retina over the course of 4 to 7 days. In a subsequent *in vitro* preparation of retina, including the retinal pigment epithelium (RPE) and choroid, the rhodamine dextran was seen to be sequestered within the cytoplasm of ganglion cell bodies, but if, under visual inspection, an individual cell was briefly exposed to light, then the tracer was liberated and spread throughout all the processes of the cell. The tracer did not appear to impair neuronal function, and the responses of the cell could be examined with an extracellular microelectrode, while visual stimuli were delivered via the microscope used for selecting cells and placing the electrode (64).

Peng et al. have linked their single-cell RNA analyses to morphology by combining fluorescent *in situ* hybridisation (“FISH”) with sparse viral labelling (19). By this means, they were able to confirm—for ON- and OFF-midgets and ON- and OFF-parasols—the tentative identifications that had emerged from their analyses of RNA patterns.

### Types of Retinal Ganglion Cells

Although there is some degree of consensus on major retinal ganglion cell types including midget RGCs, parasol RGCs and small bistratified RGCs, there has been some debate over classification of the remaining types. The functions of the remaining RGCs have been inferred from animal studies (Table 4).

**Table 4:** The classification of retinal ganglion cells in primates.

	**Stratification**	**Dendritic field size (μm)**	**Function**
Midget	Inner (above the axon terminals of DB6 bipolar cells) Outer (CD15-labeled OFF midget bipolar cells)	10–100 μm	Colour (red-green)
Parasol	Inner (above the DB6 cells) Outer (at the level of the calbindin-labelled DB3a cells)	30–300 μm	Movement
Small bistratified	Inner (above the level of DB6 axons) Outer (near or above the level of DB3a axons)		Colour (short-wave ON)
Large bistratified	Inner/Outer		
Smooth monostratified	Outer	Fewer, straight dendrites 250–340 μm	
Narrow thorny (outer/inner stratifying)	Outer (calbindin-labelled DB3a cells)/Inner (DB6 cells)	190–300 μm	
Broad thorny	In the middle of IPL (DB3a cells to CD15-labeled DB6 cells)	170–600 μm	Local edge detectors?
Recursive bistratified	DB6 cells		ON-OFF direction
Recursive monostratified			
Large sparse		240–333 μm	
Giant sparse	Bistratified (65) (Inner / Outer)	441–533 μm	

*Nomenclature by Masri et al. (8)*.

#### Midget RGCs (P-Cell, mRGCs)

This major cell type accounts for 70% of RGCs. The midget RGCs have a small-sized body with small fields of dendrites (5–10 μm in diameter in the central retina and up to 225 μm in the periphery), which correspond to smaller receptive fields than those of other RGCs. These cells are located mainly in the central retina and project to the parvocellular pathway (66, 67). Midget RGCs have a one-to-one connectivity with midget bipolar cells, which draw their input from a single cone (68). There are two types of midget RGCs: the outer stratified OFF-midget cells show smaller dendritic fields and higher cell densities than the inner ON-midget cells.

The parvocellular pathway is dominated by midget RGCs. Functional assessments of these cells demonstrate that their luminance contrast sensitivity is lower than that of parasol RGCs and most show clear chromatic opponency (69). In general, midget cells have red–green opponency, parasol RGCs are achromatic, and bistratified ganglion cells connect with S-cone ON and L-M cone OFF pathways. However, recent studies suggest that some OFF-midget cells receive signals from short wavelength (blue) sensitive cones (14, 70). Electron microscopy reconstructions of retinal circuits suggest the possibility that a small proportion of midget ganglion cells might have blue–OFF, yellow–ON receptive fields. In addition to colour discrimination, midget RGCs also subserve pattern, texture and stereoscopic depth perception (71).

#### Parasol RGCs (M-Cell, pRGCs)

Parasol RGCs project to the magnocellular layer of the LGN. As with midget cells, there are two types of parasol cells in primates: ON-parasol cells respond with an increase in firing rate at the onset of light in the centre of their receptive field whereas OFF-parasol cells react to off stimuli (63). In synaptic connexions between ON-centre parasol cells and other cells, ~20% of the input is from bipolar cells and the remainder of the signal is introduced from amacrine cells (72).

Parasol RGCs have larger receptive fields and cell bodies, have higher sensitivity to luminance contrast, and present little or no chromatic antagonism, in contrast to midget RGCs (73). Parasol RGCs play a role in motion perception, flicker perception and depth processing based on motion parallax (71). They largely comprise the magnocellular pathway.

#### Small Bistratified RGCs

This cell type accounts for ~5–8% of RGCs (8). The small bistratified RGCs (sbRGCs) project to the koniocellular layers of the LGN. Small bistratified cells have branches in both layers (inner ON- IPL and outer OFF-IPL): inner ON-IPL branches receive excitatory input from S-ON bipolar cells initiated by S-cones, while opposed (L+M)-OFF light responses arrive through outer OFF-IPL branches (63). This arrangement is thought to give good colour vision with low spatial resolution.

#### Large Bistratified RGCs

The inputs of large bistratified RGCs have not been elucidated. Large bistratified cells may receive not only S-cone ON-pathway input, but also (L+M) cone OFF-opponency (inhibitory) signals. However, the precise role of this cell type is not yet clear (74).

#### Smooth Monostratified RGCs

Smooth monostratified RGCs (smRGCs) have irregular receptive fields with multiple distinct hotspots of light sensitivity. These cells again can be divided into ON- and OFF-cells (75). They might contribute to signalling spatial information via a non-linear mechanism, whereby output is not linearly related to input (76).

#### Recursive Monostratified/Bistratified RGCs

The recursive RGCs have moderately densely branched dendritic trees in which many secondary branches tend to curve back towards the soma or close loops of apposing and recursive dendrites. In addition, many dendrites overlap those of neighbouring cells (77). These features resemble those of the directionally selective, motion-sensitive RGCs (dsRGCs) of the rabbit, in which seven types of dsRGCs have been described, namely, ON-types specific to three different directions and ON-OFF types, specific to four different directions. To date, only one population of a bistratified ON-OFF type has been described in the macaque retina (78–80).

#### Thorny RGCs

There are three types of thorny RGCs in the primate retina that account for ~1% of ganglion cells (77). Broad thorny RGCs are given various names such as thorny diffuse, T-group cells, S3 narrow thorny, and hedge cells (30, 49, 81). The dendrites of broad thorny RGCs span a whole layer of the inner plexiform layer. It is presumed that the cells may contribute to ON/OFF-centre light responses that are strongly suppressed by stimulation of the receptive field surround, such as local edge detector cells in rabbits (82). Additionally, two narrowly stratified cells, including outer and inner, are found in primates and their connectivity has not been clarified yet.

#### Large Sparse RGCs

These cell types are monostratified cells that receive input from amacrine and bipolar cells. The transcription factor Satb2 is expressed in large sparse RGCs in macaque and human retina (83).

#### Melanopsin-Containing Intrinsically Photosensitive RGCs (ipRGCs)

**Figure 4:**
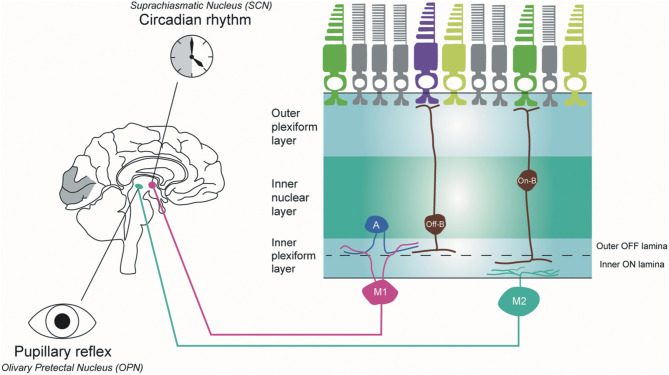
Melanopsin-containing intrinsically photosensitive RGCs in primates. Inner stratifying ipRGCs (M2 ipRGCs) have larger soma and more branched dendrites than outer stratifying ipRGCs. In addition, the dendrites of M1 ipRGCs are mainly located in the outer IPL layer, whereas M2 ipRGCs are in the outer IPL layer close to the ganglion cell layer. M1 ipRGCs project to the suprachiasmatic nucleus (SCN) to synchronize the circadian clock and M2 ipRGCs project to the olivary pretectal nucleus (OPN) in the thalamus to control pupillary response. A, amacrine cell; Off-B, Off-bipolar cell; On-B, On-bipolar cell (*by Ungsoo S. Kim*).

The ipRGCs constitute 1% of the total RGC population in humans (84). These cells have large, sparse dendritic fields. They are intrinsically photosensitive because of the expression of the melanopsin photopigment and capable of phototransduction independently of rods and cones (84). In mouse retinas, six subtypes (M1, M2, M3, M4, M5, and M6) of ipRGCs have been identified with distinctive anatomical and functional properties (Figure 4) (85). M1 and M2 ipRGCs account for the majority (74–90%) of ipRGCs. The main function of ipRGCs, in particular of the Brn3b-M1subtype, is to contribute to circadian photoentrainment through the projections to the suprachiasmatic nucleus (SCN) of the hypothalamus (86), but they are also relevant for other non-image forming functions of the eye, including the regulation of the pupillary light reflex through the projections to the OPN. M1 and M2 ipRGCs project to both the SCN and the olivary pretectal nucleus (OPN); however, M1 ipRGCs innervate the outer shell region of the OPN, where projection neurons that innervate the pre-autonomic Edinger-Westphal nucleus reside, whilst M2 ipRGCs innervate the OPN central core (87). The input dendrites of M2 and M4 ipRGCs are in the inner retina (ON-pathway), whereas those of M1 ipRGCs are located near the inner nuclear layer (OFF-pathway). The dendrites of M4 and M5 ipRGCs are located in the inner lamina of the inner plexiform layer. M4 ipRGCs have a larger cell body compared with M5 ipRGCs that have small, highly branched dendrites arrayed uniformly around the soma.

In humans, three ipRGC subtypes (M1, M2, and M4) have been defined (88). M1 ipRGCs have outer stratifying dendrites with a few smooth spines in the outer IPL, whereas M2 ipRGCs stratify in the inner part of the inner plexiform layer (IPL) close to the ganglion cell layer. M1 ipRGCs have been divided into two subtypes, gigantic M1 RGCs (GM1 cells) with round or oval large soma and displaced M1 RGCs (DM1 cells). Both ipRGC types receive inputs from DB6 bipolar cells and project to the dorsal LGN (89). M2 ipRGCs have larger soma and more branched dendrites than M1 ipRGCs. M1 ipRGCs are reported to receive an inhibitory input from short-wave cones via an S-cone amacrine cell (90), whereas M2 ipRGCs receive input from S-On bipolar cells and contribute to the blue cone pathway (91). M1 ipRGCs project to the SCN to synchronise the circadian clock and M2 ipRGCs project to the OPN in the thalamus to control pupillary response.

The ipRGCs are relatively preserved in the mitochondrial optic neuropathies, such as Leber hereditary optic neuropathy (LHON) and autosomal dominant optic atrophy (ADOA). However, ipRGCs are affected in other optic neuropathies, such as glaucoma, and late-onset neurodegenerative disorders, such as Alzheimer disease and Parkinson disease (92–95).

#### Miscellaneous RGCs

There are a small number of unclassified RGCs in primates that do not fit with any of the previously described RGCs (8). Further work is needed to elucidate the characteristics of this miscellaneous group of RGCs.

### Clinical Aspects of RGC—Methods of Assessment and Clinical Entities

#### Clinical Structural and Functional Assessment of RGCs

Although RGCs have been extensively studied in primates, the clinical assessment of RGCs has proven more challenging as they cannot be evaluated directly.

#### Structural Quantification of RGCs

##### Optical Coherence Tomography

Optical coherence tomography (OCT) is a non-invasive imaging technique that uses low-coherence light waves to capture a cross-section of various tissues. Major advances have led to the development of spectral domain OCT, which can produce a segmentation of ten layers of retina, including the retinal nerve fibre layer (RNFL) and ganglion cell layer. OCT has become a standard tool to investigate changes with RGCs as it is non-invasive, rapid, highly reproducible (96–98).

The RNFL can be measured in both the peripapillary and the macular areas. Several studies suggest that changes can be detected earlier by assessing the thickness of the RNFL in the macula compared with the peripapillary RNFL, owing to the latter's thickness (99, 100). There is a good correlation between RNFL thickness and both visual acuity and visual field changes, offering an objective structural parameter for assessing glaucoma and other optic neuropathies (101–103). However, to avoid misinterpretation of OCT, several factors need to be considered: segmentation errors can occur particularly in the presence of a tilted optic disc (104); and RNFL thickness is also affected by age as well as by refractive error and axial length. In addition, there is lag time before any changes in the thickness of the RNFL can be detected after disease onset (105), and the thickness can be overestimated in the presence of optic disc or RNFL swelling.

In addition, RNFL thickness exhibits a floor effect that must be considered in advanced optic neuropathies. RNFL thinning reaches a trough at a certain level owing to residual tissues such as vessels and glial cells (106, 107). Furthermore, RNFL loss usually signifies irreversible damage and functional tests (as described below) might be needed to identify ganglion cell dysfunction at a potentially reversible stage. It is well-established that visual acuity and visual fields can recover despite extensive RGC layer thinning (108, 109).

Microcysts in the inner nuclear layer have been reported on macular OCT imaging in some patients with advanced loss of macular RGCs. These are thought to arise from retrograde transsynaptic degeneration and/or vitreous traction in the presence of RGC and RNFL loss (110, 111). They do not seem to be specific to a particular aetiology, having been reported in patients with inherited optic neuropathies, demyelinating optic neuritis, compressive and nutritional optic neuropathies, endemic optic neuropathy and advanced glaucoma (112–114). It is not clear why these microcysts develop in only a subgroup of patients. They are seen more often in younger patients who may have a more adherent vitreous surface and ILM tension has been implicated as part of the pathophysiology (110). However, microcysts have also been reported as a long-term consequence associated with RGC loss in patients with silicon oil-related visual loss (115). These patients have undergone prior removal of the vitreous suggesting that simple vitreous traction may not be sufficient to explain the development of these microcysts.

##### Detection of Apoptosing Retinal Cells

The detection of apoptosing retinal cells (DARC) is a new technique that enables visualisation of real-time RGC apoptosis using fluorescently-labelled annexin A5. This 36 kDa protein is expressed in humans and it is a well-established indicator of apoptosis (116)). DARC has the advantage of early detection of RGC loss before visual deterioration has occurred, and it being considered for the evaluation of optic neuropathies, including glaucoma disease progression (117).

#### Functional Evaluation of RGCs

A number of psychophysical measurements can be used to investigate changes in RGC function.

##### Visual Acuity Tests

Visual acuity has been defined as the “spatial resolving capacity” of the visual system and it is the most common primary outcome measure in clinical trials. Although Snellen charts are widely used, the LogMAR scale based on the Early Treatment Diabetic Retinopathy Study (ETDRS) chart is the gold standard for clinical trials, overcoming many of the limitations of Snellen charts. However, as visual acuity tests central foveal function, patients can have widespread ganglion cell loss with preserved central visual acuity.

##### Spatial and Temporal Contrast Sensitivity Tests

Achromatic stimuli of low and high spatial frequencies can be used to differentiate responses from the magnocellular and parvocellular systems. The magnocellular pathway has lower spatial resolution and responds to higher temporal frequencies than the parvocellular pathway (118). However, this difference is relatively small and the two pathways have a degree of overlap.

##### Colour Vision Tests

Colour vision impairment is a frequent feature of ganglion cell pathology, but outer retinal dysfunction can also affect colour vision, such as anomalies of the cone photoreceptors. Congenital stationary red-green colour deficiencies commonly affect men, owing to loss or alteration of the long or medium wavelength opsin genes on the X-chromosome (119). Rarely, abnormalities in the same genetic region can give rise to S-cone monochromacy. Congenital tritan anomalies, arising from abnormalities in S-cones are also rare. Progressive or later onset cone or macular dystrophies, or congenital achromatopsia, will also affect colour vision, but in these conditions visual acuity is also usually impaired (120). In acquired ganglion cell pathology, however, visual acuity can be preserved with colour vision being preferentially affected. Many optic neuropathies affect red-green discrimination, although glaucoma commonly affects the blue-yellow axis (120).

Colour vision tests are widely used to screen patients with congenital colour vision defects and to investigate acquired pathology. There are three broad types of colour vision tests in practice (121). Pseudoisochromatic tests, such as the Ishihara, the Hardy-Rand-Rittler (HRR), and the Standard Pseudoisochromatic Plates (SPP), the Colour Vision Testing Made Easy (CVTME), and the Cambridge Colour Test are widely used. In arrangement tests, such as the Farnsworth-Munsell (FM) Dichotomous D-15 tests and 100-hue test, the patient is required to arrange a set of colours in order. The FM 100-hue test is highly sensitive, but time-consuming. Lastly, anomaloscopes are based on colour-matching where the observer adjusts a mixture of red and green lights to match a monochromatic orange light.

As congenital anomalies of colour perception more commonly affect red-green discrimination, many standard tests such as the Ishihara plates and the Nagel anomaloscope do not probe for tritan disorders, which are common in acquired pathologies. Tritan defects can be identified readily by other tests, including the D-15 and FM 100-hue, the Cambridge Colour Test, and the HRR plates. In addition, more specialised psychophysical methods, including measurement of the three primary colour vision mechanisms, colour adaptometry, and colour perimetry can be applied (122). Among them, SWAP, a specialised type of perimetry, can also be considered a colour vision test, as the targets are short-wave and the field is of long wavelength and high intensity (in order to adapt the long- and middle-wave cones) (123).

##### Visual Field Tests

In addition to conventional visual field testing, short wavelength automated perimetry (SWAP) probes the small bistratified ganglion cells and the konioceullar pathway, and high-pass resolution (ring) perimetry tests the parvocellular pathway, whereas flicker perimetry, motion perimetry, and frequency doubling technology (FDT) target the magnocellular pathway (124). Among these tests, SWAP and FDT are available as commercial products.

(1) Frequency Doubling Technology (FDT)

FDT has the advantage of greater sensitivity, potentially detecting RGC damage earlier than standard automated perimetry (SAP) (125). Modern FDT uses targets of low spatial frequency that flicker at a high temporal frequency and that predominantly stimulate the magnocellular pathway, which corresponds to motion detection and flicker detection (126). FDT has been put forward for the early detection of glaucoma on the basis that the magnocellular pathway is more vulnerable in glaucoma (127, 128). However, there is evidence that both the parvocellular and magnocellular pathways are affected early in glaucoma with no significant differences between these two pathways in terms of their vulnerability (129). Furthermore, a recent study indicated that FDT is neither sensitive nor specific as a screening tool for glaucoma (130). Further studies are, therefore, needed to evaluate the role of FDT in the early detection of glaucoma.

(2) Short Wavelength Automated Perimetry (SWAP)

Unlike standard visual field testing, which uses a white stimulus on a white background, SWAP employs a blue stimulus on a yellow background. Several studies suggested that SWAP is more sensitive for the early detection of glaucomatous changes compared with standard visual field testing (131–133). There is, however, no definitive evidence that the small bistratified ganglion cells (short-wave response) are more vulnerable in glaucoma. SWAP was reported to be 10–20 times more sensitive than standard perimetry in patients with ADOA (134). As a result, SWAP was able to differentiate between normal tension glaucoma with or without *OPA1* polymorphism (135). However, SWAP has some limitations as it is time-consuming, it needs a higher level of cooperation, and it has lower reproducibility compared with standard perimetry (136).

##### Chromatic Pupillometry

The primate pupil responds to signals from ipRGCs, which additionally receive input derived from cone responses. Chromatic pupillometry uses selective wavelengths to quantify pupil size before, during, and after a light stimulus has been applied. Comparison of pupillary responses to short-wavelength and long-wavelength light can selectively probe the function of outer retinal photoreceptors or the intrinsic response of ipRGCs. The ipRGCs are blue light sensitive and maximally sensitive to wavelengths that lie between the peak sensitivities of the rods and S-cones. Several studies using chromatic pupillometry in experimental animal models have shown that the light sensitive ipRGCs were spared in retinitis pigmentosa characterised by marked photoreceptor loss (137). Generally, the ipRGCs are relatively preserved in mitochondrial optic neuropathies, such as LHON and ADOA (138, 139), but affected in other optic neuropathies such as glaucoma, non-arteritic anterior ischemic optic neuropathy and demyelinating optic neuritis (140). Bichromatic pupillometry has been used to differentiate between mitochondrial and non-mitochondrial optic neuropathies (94, 140).

##### Electrophysiological Tests

Electrophysiology allows direct objective assessment of electrical responses *in vivo*. The visual evoked potential (VEP), recorded over the visual cortex, has long been used as a means of assessing the function of the visual pathway, as well as demonstrating developmental abnormalities, such as the misrouting of ganglion cell axons in albinism (141). In addition, the electroretinogram (ERG), which represents the summed electrical response of the retina to light stimuli, can be recorded non-invasively. The pattern ERG (PERG), arising from stimulation of the macula, is largely derived from responses in the macular RGCs. In contrast, the full-field ERG, which is generated from the stimulation of the whole retina, is usually used to evaluate responses from photoreceptors and bipolar cells. However, a late component, the photopic negative response (PhNR) has been shown to arise in ganglion cells.

(1) Pattern Electroretinogram

The PERG is recorded in response to a patterned stimulus (typically a checkerboard pattern reversing 4 times per second), which stimulates the central 15 degrees of the retina (142). The PERG comprises a cornea-positive wave at 50 ms (termed P50) and a negative wave at 95 ms (termed N95). The test is performed in photopic conditions with undilated pupils and it requires optimal refraction. The response is driven by the macular cone photoreceptors, but it appears to arise largely from the macular RGCs, whose signals appear to give rise to the N95 component and the majority of the P50 component (143, 144). Various optic neuropathies that affect the ganglion cells within the retina (either as the primary site of impairment or from retrograde degeneration from an optic nerve lesion), for example demyelinating optic neuritis, ischemic optic neuropathy, compressive optic neuropathy, toxic optic neuropathy, and inherited optic neuropathies can result in a reduction of the N95 and P50 amplitudes, with N95 being reduced more than P50, and a shortening of the P50 peak time (145–147). Whilst the PERG is sensitive to macular RGC dysfunction, precise correlation with RGC subtype is not known, and the test will not detect extramacular RGC impairment.

(2) Photopic Negative Response

The PhNR is a negative wave of long latency that follows the b-wave of the photopic cone-driven ERG and it arises in RGCs (148). Whilst it can be detected in standard white-on-white flash responses, specific chromatic protocols can be used to optimise the PhNR signal (149). As with the PERG, the amplitude of the PhNR decreases in optic nerve disorders (150, 151). Unlike in PERG recordings, optimal refraction is not needed, but the pupils need to be dilated. In addition, a hand-held mini-Ganzfeld stimulator is available to test PhNR (152). The flashes stimulate the retina as a whole so the PhNR can be indicative of global RGC function.

Focal PhNR recordings can be performed to assess RGCs over a particular region (typically the macula) (153). The PhNR can be used to examine the parvocellular pathway whereas the steady-state PERG is focused on the magnocellular pathway in glaucoma (154). Although the PERG and PhNR can detect glaucoma, there is no significant correlation between PhNR ratio and PERG ratio values (155).

### Clinical Correlates—Structure and Function

**Figure 5:**
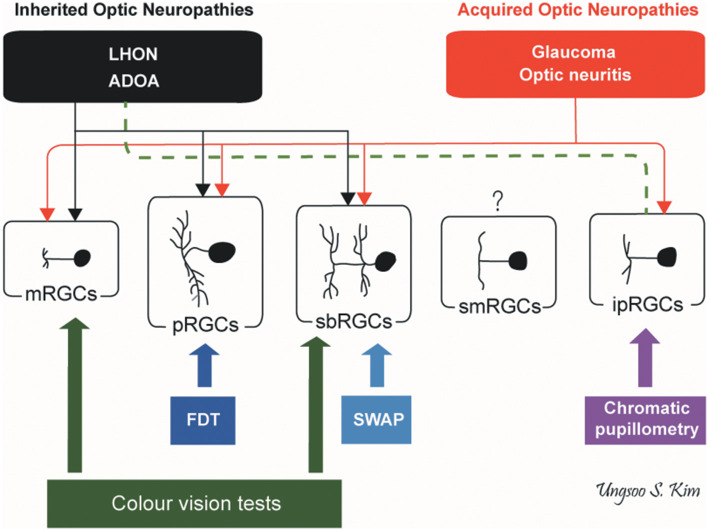
Pattern of RGC involvement in optic neuropathies. The types of RGCs affected in inherited optic neuropathies and acquired optic neuropathies are indicated by black and red lines, respectively. The dotted green line indicates the preservation of ipRGCs in inherited optic neuropathies. ADOA, autosomal dominant optic atrophy; FDT, frequency doubling technology; ipRGCs, intrinsically photosensitive retinal ganglion cells; LHON, Leber hereditary optic neuropathy; mRGCs, midget retinal ganglion cells; pRGCs, parasol retinal ganglion cells; sbRGCs, small bistratified retinal ganglion cells; smRGCs, smooth monostratified RGCs; SWAP, short wavelength automated perimetry (*by Ungsoo S. Kim*).

Inherited and acquired optic neuropathies are important causes of registrable blindness. Treatment options remain limited, and when available, they mostly slow down or prevent further loss of RGCs. Visual loss is usually irreversible although in some cases, spontaneous visual recovery can occur owing to the functional recovery of RGCs that have not undergone apoptosis. To better inform future treatment strategies, it is essential to gain a better understanding of the pattern of RGC loss and whether different aetiological triggers result in global or more selective loss of RGCs, and how these relate to the visual deficits and eventual outcome. It remains a challenging task as patients are not always examined in the acute stage of the disease and serial measurements are needed to document progression over time. Nevertheless, we are gaining a better understanding of the structure-function relationship in different optic neuropathies aided by the availability of high-resolution retinal imaging with OCT and more sophisticated visual electrophysiological and psychophysical tools (Figure 5).

### Inherited Optic Neuropathies

The minimum prevalence of inherited optic neuropathies has been estimated at 1 in 10,000 (156). This group of disorders is genetically heterogeneous with disease-causing mutations occurring in both mitochondrial and nuclear DNA (157). Remarkably, all genes identified to date encode proteins that are either directly or indirectly involved in regulating mitochondrial function. The generation of ATP by the mitochondrial respiratory chain is central to cell survival and mitochondria also regulate other key pathways, including the level of reactive oxygen species and the tight control of apoptosis. An intriguing aspect of inherited optic neuropathies is the preferential vulnerability of RGCs compared with other neuronal populations despite the ubiquitous expression of the genes involved. There have been limited post mortem studies on the pattern of RGC loss in inherited optic neuropathies owing to the lack of access to diseased human tissues. Nevertheless, useful insight has been obtained with the application of high-resolution OCT imaging and psychophysical evaluation of patients at different stages of the disease process. The two best studied inherited optic neuropathies are LHON and ADOA.

(1) Leber Hereditary Optic Neuropathy

LHON is a primary mitochondrial DNA (mtDNA) disorder and ~ 90% of cases are due to one of three mtDNA point mutations, namely, m.3460G>A (*MT-ND1*), m.11778G>A (*MT-ND4*), and m.14484T>C (*MT-ND6*) (158, 159). The peak age of onset is from 15 to 35 years old and the majority of patients are men (80–90%) (160). Although bilateral simultaneous onset can occur in some patients, sequential involvement of the second eye within a few months is more typical. LHON is characterised by severe visual loss with dyschromatopsia and a dense central or cecocentral scotoma on visual field testing. OCT initially shows swelling of the RNFL, follows by marked thinning of RNFL, especially in the temporal quadrant corresponding to the papillomacular bundle (161). Childhood-onset LHON and the m.14484T>C mutation are associated with a more favourable visual outcome (38, 162). Most patients with LHON are registered legally blind with <20% of patients carrying the m.11778G>A mutation experiencing some visual recovery (159, 163).

In LHON, RGCs with the smallest calibre axons, which have smaller mitochondrial reserve per energy requirement, are preferentially affected and these are predominantly located within the papillomacular bundle (164, 165). The peripapillary RNFL is swollen in the acute stage of LHON, as demonstrated by OCT, with subsequent thinning occurring as the disease progresses into the chronic stage. Measurement of ganglion cell and inner plexiform layer (GC-IPL) thickness in the macular area indicate that pathological thinning is already evident in the pre-symptomatic stage about 6 weeks before the onset of visual loss in the fellow eye (166). These findings suggest that midget RGCs, which are a major component of the papillomacular bundle, could be more vulnerable to the underlying mtDNA mutation. Selective attenuation of four of the six layers in the LGN that are connected to the parvocellular pathway have been reported, but this feature is controversial as the magnocellular pathway is known to be also affected in LHON (167, 168). Further investigations are needed to determine the primary defect.

The ipRGC subtype is relatively preserved in LHON, explaining why the pupillary light reflex is maintained even in severely affected patients (138). The mechanisms that account for this enhanced resilience of ipRGCs remain unclear, although several hypotheses have been proposed (140). From an anatomical perspective, ipRGCs are predominantly located in the parafoveal area and at the far end of the nasal hemiretina, rather than feeding into the papillomacular bundle (92). In a post mortem study of a patient carrying the m.3460G>A mtDNA mutation, the pupillary fibres in the pretectum were found to be preserved (169). It is possible that ipRGCs are protected because of their higher concentration of mitochondrial cytochrome *c* oxidase and a greater abundance of mitochondria (140). Several protective factors such as PI3K and pituitary adenylate cyclase-activating polypeptide (PACAP) may further reinforce the survival of ipRGCs under certain conditions (170, 171).

(2) Autosomal Dominant Optic Atrophy

ADOA is the most common inherited optic neuropathy with an estimated prevalence of 1 in 25,000 in the general population (172). Mutations in the nuclear gene *OPA1* (3q28-q29) account for ~70% of all cases of ADOA (173). The classical clinical features of ADOA are progressive bilateral visual loss starting in early childhood, dyschromatopsia, a central or cecocentral scotoma, and optic disc pallor that is more prominent temporally due to the preferential involvement of the papillomacular bundle (174). There is a marked variability in disease severity with visual acuity ranging from 6/6 to light perception, and variable rates of disease progression even within the same family (175). OCT typically shows RNFL thinning, which is more marked temporally, with gradual loss of RGCs occurring over time (176). The disease process is thought to start *in utero* with *OPA1* carriers having a reduced number of RGCs at birth compared with normal healthy individuals (138).

In ADOA, midget RGCs, parasol RGCs and small bistratified RGCs are all affected, impairing sensitivity to high spatial frequencies, long- and middle-wave colour discrimination, sensitivity to high temporal frequencies, and short-wave sensitivity. The S-cone–related losses showed a significant deterioration with increasing patient age and could therefore prove useful biomarkers of disease progression in ADOA (177). The S-cone chromatic response and koniocellular pathway are impaired in the early stage of the disease, which suggest a vulnerability of small bistratified RGCs (173). Although tritanopia has been reported as the characteristic colour vision defect in ADOA, only 7.5% of patients with ADOA had exclusively tritanopia in one study, with the most common colour defect in 81.2% of patients being of the mixed type (134).

As in LHON, the pupillary response in ADOA is relatively preserved, indicating that ipRGCs in mitochondrial optic neuropathies appear to be more resistant to the underlying mitochondrial dysfunction compared with other RGC subtypes. Studies using chromatic pupillometry also reported preservation of ipRGCs in ADOA patients with severe visual loss and optic atrophy (178, 179).

#### Acquired Optic Neuropathies

There is a long list of aetiological factors that can result in RGC injury and optic nerve degeneration. Compared with inherited optic neuropathies, fewer studies have focused specifically on RGC pathophysiology in acquired optic neuropathies. More work is, therefore, needed to elucidate subtype selectivity, if any, of RGC loss in ischemic, compressive, inflammatory, autoimmune and paraneoplastic optic neuropathies. However, we do know that most toxic optic neuropathies have an underlying mitochondrial aetiology (180). There is a growing body of evidence that mitochondrial dysfunction plays a prominent pathophysiological role in glaucoma, demyelinating optic neuritis and toxic optic neuropathies (181, 182). This aetiological link is relevant and comparing the pattern of RGC loss between these acquired optic neuropathies and classical monogenic optic neuropathies could reveal common pathways amenable to therapeutic intervention.

(1) Glaucoma

Glaucoma is a leading cause of irreversible blindness affecting 3–5% of the population over the age of 70 years (183). Extrafoveal RGCs usually deteriorate in the early stages resulting in arcuate scotomas in the visual field. Traditional anatomical studies reported greater loss of axons of large diameter, corresponding to the magnocellular pathway (parasol cells) (184), and the magnocellular LGN layers were more affected compared with the parvocellular LGN layers (185). However, there are rarer types of retinal ganglion cells with large axons and further investigations are needed to evaluate the changes of these cells in glaucoma. The relative vulnerability of large axons in glaucoma may simply reflect the anatomical location of the affected ganglion cells. Glaucoma patients have poor response to high temporal frequency light stimuli that correspond to the magnocellular pathway. In a primate study using immunohistochemistry, a decrease in large RGCs was observed after elevating IOP (186). This specific vulnerability was ascribed to calcium-permeable receptors, the relative proximity of RGCs and their dendrites to blood supply in the IPL layer, and the differing metabolic requirements of these particular large cell types (187). However, other studies suggested no predilection for a specific pathway (188, 189). Compared with inherited optic neuropathies, the ipRGCs are vulnerable in both patients with confirmed glaucoma and glaucoma suspects (190, 191). In contrast, ocular hypertension does not seem to result in significant loss of ipRGCs (192).

(2) Demyelinating Optic Neuritis

Inflammatory demyelination resulting in optic neuritis is a major manifestation of multiple sclerosis. Inflammation of the retinal vascular endothelium can precede demyelination and perivascular cuffing and oedema of the optic nerve sheath leads to breakdown of myelin (193). Idiopathic demyelinating optic neuritis leads to visual loss with minimal axonal loss.

Optic neuritis is associated with alteration of both the parvocellular and magnocellular pathways (194). Viret et al. suggested that the more heavily myelinated magnocellular axons are more vulnerable in patients with optic neuritis because low spatial frequencies, which are transmitted by the magnocellular pathway, are affected predominantly 1 month after the acute phase of the optic neuritis episode (195). Despite the recovery of visual acuity, the magnocellular pathway did not fully normalise (196). In contrast, a significant loss at high spatial frequencies has been reported in the affected eye and the parvocellular pathway was more impaired in patients with resolved optic neuritis who had 20/20 visual acuity after recovery (197). Fallowfield and Krauskopf suggested that chromatic discrimination is more severely impaired than luminance discrimination in the demyelinating diseases (198). This discrepancy might be due to differences in the timing and severity of optic neuritis. Consequently, it is still unclear which pathway is more vulnerable in the context of demyelinating optic neuritis (196). Both red-green and tritan defects have been reported in optic neuritis (199). Characteristics of colour deficiency may change over time as assessed with the FM 100-hue test, with blue-yellow defects being more common in the acute stage and red-green changes being predominant in the chronic stage (200). It is possible, of course, that the variability of symptoms in optic neuritis reflects immunologically distinct conditions that differentially affect different types of RGCs.

(3) Toxic Optic Neuropathy

Various substances such as ethambutol, isoniazid, linezolid, chloramphenicol and methanol can cause optic nerve dysfunction, probably through acquired mitochondrial dysfunction (180). As in inherited optic neuropathies, the papillomacular bundle is selectively vulnerable and this typical feature can be confirmed by optical coherence tomography, which shows a profound decrease in temporal RNFL thickness. The parvocellular pathway within the papillomacular bundle is affected extensively likely secondary to a number of factors, including smaller and more thinly myelinated nerve fibres and a faster firing response with higher average rates of action potentials (201). However, there is a lack of evidence on whether this is simply because the parvocellular neurons predominate in the papillomacular bundle, or whether the midget cells are the primary target of the triggering toxic substances.

#### Clinical Relevance and Future Work

The physiological features of the major RGC subtypes (mRGCs, pRGCs, and sbRGCs) are well-known, but the role and characteristics of other RGCs require further study. An in-depth characterisation of the chronological structural and functional changes occurring within the RGC layer in optic nerve disorders, including inherited and acquired optic neuropathies, are important to inform the future design of clinical trials. Understanding which RGC subtypes are selectively affected will help optimise outcome measures in natural history studies and trials of experimental therapies. As mentioned earlier, the FDT test is used for the early detection of glaucoma because the magnocellular pathway is more vulnerable (127). Given that a common variant in the *SIX6* gene (rs33912345) is strongly associated with primary open-angle glaucoma (POAG) and the fact that this gene is highly expressed in midget RGCs, tests that evaluate this particular cell type could prove to be a useful sensitive biomarker of disease progression (19, 202).

The remarkable advances in gene delivery and editing technology have led to an increasing number of clinical trials for optic neuropathies, in particular gene replacement therapy for monogenic inherited optic neuropathies (203). Gene therapy using adeno-associated viral vectors is currently favoured and there is now cumulative evidence of its long-term safety and efficacy in delivering gene constructs to retinal cells (204, 205). Promising results have been obtained with allotopic expression of the *MT-DN4* gene in patients with LHON treated within 1 year of disease onset (206, 207). To enhance success of gene therapy, optimised tissue-specific promoters, which control expression of the therapeutic gene, are needed, and these could potentially. be optimised for the relevant RGC subtype (208). Genomic editing, such as the CRISPR-Cas system, and stem cell therapy is an exciting development that has the potential to revolutionise the treatment of ophthalmological disorders given the eye's relative ease of anatomical access and its relative immune privilege (209, 210). The intriguing preservation of ipRGCs in mitochondrial optic neuropathies needs to be investigated further as the factors that confer this resilience would be obvious therapeutic targets (211).

There is increasing interest in employing RGCs to restore visual function in the retinal dystrophies marked by widespread loss of rods and cones (212). Optogenetic therapies are being developed to confer light sensitivity to inner retinal neurons, which are spared in these forms of outer retinal degeneration. Another approach is the use of electronic implants to stimulate these inner retinal neurons so that visual signals can be transmitted to the brain. A better understanding of inner retinal connectivity, specifically that of RGCs, is essential to optimise these innovative sight restoring strategies. Elucidating the selective vulnerability of RGCs compared with other retinal and neuronal cell types in inherited optic neuropathies is key to developing targeted treatments for this group of disorders. The availability of high-throughput transcriptomic techniques that can be conducted at the single cell level is an exciting development, providing us with powerful tools to identify pathways that can be modulated for generalizable, mutation-independent neuroprotective strategies (213). Although appealing, regenerative medicine will require not only the replacement of the missing RGCs, but also the establishment of the sophisticated circuitry that allows the integration of signals from various pathways to achieve a reasonable degree of visual perception (214).

## Conclusion

Ganglion cells constitute the output pathway of the retina, transmitting highly processed and integrated signals to the visual processing areas in the brain. Up to 18 types of RGCs have been reported, constituting a sophisticated repertoire of cell types each with specific attributes contributing to visual perception. Future studies will further dissect the selectivity and timing of impairment of RGC subtypes in various optic neuropathies and how these could be modulated in the context of experimental therapies. The refinement of tests to assess RGC structure and function is relevant not only for clinical practise, but also for deep phenotyping as part of natural history studies and to define relevant outcome measures for clinical trials. We are now entering an exciting translational phase for optic neuropathies with the confluence of genetic breakthroughs and targeted therapies giving hope that we will soon be able to slow or prevent the irreversible loss of RGCs in these blinding diseases.

## Author Contributions

UK, PY-W-M, and JM contributed to the conception and design of the study. UK wrote the first draft of the manuscript. UK, PY-W-M, JM, and OM wrote sections of the manuscript. All authors contributed to manuscript revision, read, and approved the submitted version.

## Conflict of Interest

The authors declare that the research was conducted in the absence of any commercial or financial relationships that could be construed as a potential conflict of interest.
